# Physicochemical quality assessment of various brands of paracetamol tablets sold in Freetown Municipality

**DOI:** 10.1016/j.heliyon.2024.e25502

**Published:** 2024-02-02

**Authors:** Ahmed Vandy, Eugene Conteh, Michael Lahai, Marie Kolipha-Kamara, Mohamed Marah, Foday Marah, Kadiatu M. Suma, Sia C. Mattia, Kenneth D.S. Tucker, Victor S.E. Wray, Abass Koroma, Aiah U. Lebbie

**Affiliations:** a Faculty of Pharmaceutical Sciences - College of Medicine and Allied Health Sciences, University of Sierra Leone, Sierra Leone

**Keywords:** Paracetamol, Physicochemical test, Pharmacopoeia, Substandard medicines, Quality assessment, Freetown

## Abstract

Paracetamol is a widely used over-the-counter drug for managing fever and pain, but its quality may vary among different brands, especially in low- and middle-income countries, where counterfeit and substandard medicines are prevalent. This study evaluated the physicochemical properties of fifteen brands of 500 mg paracetamol tablets sold in various pharmacies in Freetown, Sierra Leone using identification tests, friability tests, assay, dissolution tests, and mass variation.

The results showed that three brands were not registered with the Pharmacy Board of Sierra Leone, and two brands did not meet the requirement for labelling (no manufacturing date). All the brands met the requirement for mass variation, friability tests and assays. The percentage assay of the different brands ranged from 96.17 %w/w to 101.97 %w/w. However, two brands did not meet the specification for dissolution, with P012 releasing about 21.23 % ± 5.76 of the drug within 45min.

Most of the paracetamol brands evaluated met the physicochemical test specification. However, two brands failed the dissolution test, two brands did not meet the labelling requirement and three brands were identified as unregistered products with the National Medicines Regulatory Authority in Sierra Leone. This study underscores the necessity of enhancing monitoring and post-market surveillance of pharmaceuticals in Sierra Leone to ensure they comply with regulatory requirements.

## List of abbreviations

W.H.O: World Health Organization
BP: British Pharmacopoeia
TS: Test solution
S: Stage
SD: Standard deviation
AV: Acceptance Value
UV: Ultraviolet–visible
CSMs: Counterfeit and substandard medicines
rpm: Revolutions per minute
NLT: Not less than

## 1 Introduction

Paracetamol (Acetaminophen) is a widely used non-prescription drug for managing fever and pain due to its analgesic and antipyretic effects [1]. It can be used for mild to moderate pain, but when combined with other potent analgesics, it helps in severe pain [[2], [3]]. While paracetamol is generally safe at lower doses for reducing fever and relieving pain, it poses risks such as gastrointestinal issues, liver failure, hepatotoxicity, and centrilobular necrosis in the liver [4]. Paracetamol has been listed as one of the 200 essential medicines by the World Health Organization (W.H.O) since 1991. However, its quality may be compromised especially in low- and middle-income countries where the demand is high [5].

Counterfeit and substandard medicines (CSMs) are a major public health concern, particularly in developing countries with poor regulatory standards [6,7]. According to W.H.O., about 10 % of the global supply of medicines is falsified or substandard [5], with Africa having the highest prevalence of CSMs reported. There is no reliable data on the level of CSMs sold in Freetown, Sierra Leone, especially with the increased rate of drug peddling. Peddlers smuggle drugs into the country without being tested by regulatory bodies and thus their quality cannot be accounted for. Moreover, the main sources of drug import for most sub-Saharan African countries, the Chinese and Indian pharmaceutical industries, have been classified as the major producers of fake medicines in the world [8]. W.H.O. estimated that pharmaceutical industries are losing over USD 431 billion each year in sales to manufacturers of counterfeit medicines [9]. In 2009, a report from the Pharmacy Board of Sierra Leone stated that over USD 150 million worth of counterfeit drugs were brought into the country [10].

CSMs pose a serious threat to the health care system and the public health. They can cause a lack of trust and hope in the health care system, prolonged treatment durations, treatment failure and increased cost, toxicity and chemical-induced disease due to possible chemical reactions, and even death in severe cases. Some reported cases include, in 1990, counterfeit paracetamol led to the death of 109 children in Nigeria [[11], [12]]. In 2008, a paracetamol teething formula called “My Pekin” led to the death of over 84 children in Nigeria [13]. Paracetamol syrup tested in Pakistan contained an insignificant quantity of active ingredients [14]. The high demand for these drugs, the low risk of detection due to inadequate monitoring and regulation, and weak penalties have been identified as the main reasons why CSMs are rampant in low- and middle-income countries [8]. As such, mitigating these threats posed by counterfeit medications requires stringent regulatory monitoring, robust testing, legislative actions, and accessible platforms for reporting cases of counterfeit drugs [15].

In Sierra Leone, despite the widespread use of paracetamol tablets as an over-the-counter (OTC) drug, there is a lack of published data and scientific investigation on their quality. The borders around the country are porous and allow the entry of illegal drugs that may not be regulated by the Pharmacy Board of Sierra Leone (PBSL), which is the National Drug Regulatory Authority in Sierra Leone [16]. The Pharmacy and Drugs Act 2001 used by PBSL has existing gaps in policies and legislation which affects effective pharmacy regulation and thus requires revision [17]. These gaps create room for weak regulatory measures and the circulation of possible substandard or counterfeit pharmaceutical products in the market, posing a risk to public health [18]. Our study evaluated the physicochemical quality of fifteen brands of paracetamol using Pharmacopoeia and Non-Pharmacopoeia Test methods. This study determined the registration status, percentage assay of paracetamol content and dissolution profile using Ultraviolet–Visible (UV) spectroscopy, uniformity of dosage units (mass variation), and friability of fifteen brands of paracetamol tablets. The findings from this study will contribute valuable scientific evidence regarding the quality assessment of essential drugs, particularly in low-income countries, and serve as a foundation for informed policy recommendations and regulatory enhancements in pharmaceutical quality control.

## 2 Methods

### 2.1 Study setting, design, and period

This was an experimental study that involved laboratory analysis of different brands of paracetamol tablets 500 mg. The study was conducted from June to August 2022 at the Faculty of Pharmaceutical Science College of Medicine and Allied Health Sciences laboratory and the Physicochemical Laboratory at the Pharmacy Board of Sierra Leone.

### 2.2 Sample collection

A random selection of fifteen distinct brands of 500 mg paracetamol tablets was procured from various pharmacies within Freetown. This random acquisition took place in June 2022. These samples were labelled as P001 to P015 to avoid bias, ensure confidentiality and for easier reference.

### 2.3 Test methods

The samples were analyzed using Physicochemical parameters, which included Pharmacopoeia Tests (Identification Test II, Friability, Dissolution, Assay, Uniformity of Dosage Unit) and Non-Pharmacopoeia Tests (Visual Examination, Identification Test I) [[19], [20], [21]].

### 2.4 Visual examination

This involved inspecting the parameters such as registration status with the Pharmacy Board of Sierra Leone, product description, packaging type and integrity, physical damage and other observations of each sample.

### 2.5 Identification test

The identification tests were conducted to establish the presence of paracetamol's active ingredients in the samples. Two different identity tests were conducted.

#### 2.5.1 Identity test I

0.10g of Powdered paracetamol from each sample was dissolved in 10 mL of water and 1 drop of ferric chloride, Test Solution (TS) was added; a violet-blue colour would indicate the presence of paracetamol (Basic tests for pharmaceutical substances, WHO - [22]).

#### 2.5.2 Identity test II

0.10 g of Powdered paracetamol was boiled in 10 mL of concentrated Hydrochloric acid TS for 3 min, and 10 mL of water was added and cooled; no precipitate was formed. 1 drop of potassium dichromate TS was added; a violet colour was slowly produced which does not turn red (distinction from phenacetin), (BP Vol. 3 2017).

### 2.6 Paracetamol tablets assay

Twenty tablets of paracetamol from each brand were individually weighed and powdered. A portion of the powder, containing 0.15 g of paracetamol, was accurately weighed and transferred to a conical flask and 50 mL of 0.1 M sodium hydroxide was added. It was then diluted with 100 mL of water, shaken for 15 min in an automated shaker and diluted to 200 mL with water. The solution was filtered and 10 mL of the filtrate was diluted to 100 mL with water. Then, 10 mL of this solution was added to 10 mL of 0.1 M sodium hydroxide and diluted to 100 mL with water. The sample was then analyzed by UV spectroscopy.

The Lambda UV spectrophotometer was auto-zeroed using a blank solution of 0.1 M sodium hydroxide in a cuvette. The absorbance of the sample was measured by placing some amount of the sample solution in a cuvette and inserting it into the spectrophotometer. The measurement was done using three replicates for each sample. The paracetamol content was calculated using a value of 715 as A (1 %, 1 cm) at the maximum wavelength of 257 nm. The same procedure was repeated for each brand analyzed.

### 2.7 Mass variation

The Sartorius analytical balance was calibrated before use and ten tablets of each brand were weighed collectively on the balance and recorded in grams (g). The active substance content was calculated and expressed as the percentage of label claim of each tablet from the mass of the individual tablets and the result of the assay. The Acceptance Value (AV) was calculated using the formula below:

$$\text{Formula}\;1:\text{AV}=\left|M-\bar{x}\right|+\text{KS}$$

K = Acceptability constant in 2017 BP Pharmacopoeia, n = Sample size (10 tablets), $\bar{x}$ = Average % Assay, M = Reference value based on $\bar{x}$, S = Standard deviation

When 98.5 %≤ $\bar{x}$ ≤ 101.5 %, $\bar{x}$ = M, then AV = KS,

If $\bar{x}$ <98.5 %, M = 98.5 %, then AV = 98.5- $\bar{x}$ + KS,

If $\bar{x}$ >101.5, M = 101.5 %, then AV = $\bar{x}$ - 101.5 + KS

The Acceptance Value (A.V) was calculated according to BP Volume V, 2017: maximum allowed AV ≤15.

### 2.8 Dissolution profile

The dissolution test was performed using Agilent Dissolution Apparatus 708-DS (Paddle Apparatus). Six tablets of each brand were individually placed with 30-s intervals between each placement in six vessels containing 900 mL of phosphate buffer pH 5.8 as the dissolution medium. The apparatus was run for 45 min with the paddle rotating at 50 rpm and the temperature maintained at 37 ± 1 °C. A sample of 20 mL of the medium with dissolved paracetamol was withdrawn and filtered. The filtrate solution X, which was expected to contain about 0.00075 % w/v of paracetamol, was collected and analyzed by UV spectroscopy.

The limit % specified by BP Vol 5, 2017:

Average percentage of drug released Q (where Q = 75 %)

S1: (Q + 5 %) i.e., not less than 80 % dissolved in 45 min.

S2: An average of 12 units (S1+S2) is equal to or greater than Q, and no unit is less than Q-15 % According to BP Vol 5, 2017, if a sample fails the first stage test S1, the testing will be continued through the other stages S2, S3 as described.

### 2.9 Friability test

The Friability test measures the tendency of a tablet to chip, break or crumble after compression, physical shock or abrasion during manufacturing, packaging, shipping or handling. It is usually applied to uncoated tablets.

The friability test was performed using the Vankel Friabilator. Twenty tablets from each brand were initially weighed using an electronic scale (W1) g and transferred into the Friabilator, which was run at 25 rpm for 4 min (100 revolutions). Then, the tablets were carefully removed from the Friabilator, leaving the dust particles, and weighed again (W2) g. The percentage of friability was calculated using the following formula below:

$$\text{Formula}\;2:\text{Friability}=\frac{\text{Weight}\;\text{of}\;\text{tab}\;\text{before}\;\text{test}-\text{Weight}\;\text{of}\;\text{tab}\;\text{after}\;\text{test}}{\text{Weight}\;\text{of}\;\text{tab}\;\text{before}\;\text{test}}\times 100\;\%$$

BP Volume V, 2017 limit specification: Not more than 1 % loss of the total weight.

### 2.10 Data processing and analysis

Upon the conclusion of all the laboratory analysis, data related to each test was computed and subjected to analysis through the application of a mathematical formula, utilizing MS-Excel® version 2016.

## 3 Results and discussion

### 3.1 Visual inspection

Table 1 provides detailed information about the visual inspection test results for the different brands of paracetamol.

**Table 1:** Visual inspection results.

Brands	Product Description	Manufacturing & Expiry date	Batch no.	Manufacturing country	Registration Status
P001	Uniform white-coloured, circular-shaped tablets with a scoring at the centre. No cracks and the tablets did not break prior to removal from the blister.	Mfg. Date: 03/21 Exp. Date: 03/26	1404A	Nigeria	Registered
P002	Uniform white-coloured, oval-shaped tablets with a scoring at the centre. Smooth edges. No cracks and the tablets did not break prior to removal from the blister.	Mfg. Date: NA Exp. Date: 11/23	111220	China	Registered
P003	Uniform white-coloured, shiny, oval-shaped tablets with a scoring at the centre. Smooth edges. No cracks and tablets did not break prior to removal from blister packs	Mfg. Date: 08/21 Exp. Date: 07/25	G011332	India	Registered
P004	Dirty white Coloured, oval-shaped tablets, there is no scoring at the centre. Smooth edges. No cracks and tablets did not break prior to removal from blister packs.	Mfg. Date: 02/21 Exp. Date: 02/24	C021	Nigeria	Unregistered
P005	Uniform white-coloured, oval-shaped tablets with a scoring at the centre. Smooth edges. No cracks and tablets did not break prior to removal from blister packs.	Mfg. Date: 11/21 Exp. Date: 10/24	PNTQT-006	India	Registered
P006	Uniform white-coloured, oval-shaped tablets with a scoring at the centre. Some had rough edges. No cracks and tablets did not break prior to removal from blister packs.	Mfg. Date: 10/21 Exp. Date: 09/24	HNVQT-001	India	Registered
P007	Uniform white Coloured, oval-shaped tablets with a scoring at the centre. Smooth edges. No cracks and the tablets did not break prior to removal from blister packs.	Mfg. Date: 09/21 Exp. Date: 08/24	AI 0006	India	Registered
P008	Uniform white-coloured, oval-shaped tablets with a deep scoring at the centre. Smooth edges. No cracks and tablets did not break prior to removal from blister packs.	Mfg. Date: 09/20 Exp. Date: 08/23	MP20652	India	Registered
P009	Uniform white-coloured, circular-shaped tablets with a scoring at the centre. Smooth edges. No Cracks and the tablets did not break prior to removal from blister packs.	Mfg. Date: 11/21 Exp. Date: 10/24	M21040	India	Registered
P010	Uniform white-coloured, circular-shaped tablets with a scoring at the centre. Smooth edges. No Cracks and the tablets did not break prior to removal from blister packs.	Mfg. Date: 07/21 Exp. Date: 06/24	210716	China	Registered
P011	Uniform white-coloured, oval-shaped tablets with a deep scoring at the centre. Smooth edges. No Cracks but one of the tablets did break prior to removal from blister packs.	Mfg. Date: NA Exp. Date: 09/25	E6220021	United Kingdom	Unregistered
P012	Uniform white-coloured, circular-shaped tablets with a scoring at the centre. Smooth edges. No Cracks and the tablets did not break prior to removal from blister packs.	Mfg. Date: 03/22 Exp. Date: 02/25	T002210	India	Registered
P013	Uniform white colour, circular shape tablets with a scoring at the centre. Smooth edges. No Cracks and the tablets did not break prior to removal from blister packs.	Mfg. Date: 01/22 Exp. Date: 01/25	220104	China	Registered
P014	Uniform white-coloured, circular-shaped tablets with a scoring at the centre. Smooth edges. No cracks and tablets did not break prior to removal from blister packs.	Mfg. Date: 09/20 Exp. Date: 08/23	V20182	India	Unregistered
P015	Uniform white-coloured, circular-shaped tablets with a scoring at the centre. Smooth edges. No cracks and the tablets did not break prior to removal from blister packs.	Mfg. Date: 04/22 Exp. Date: 03/25	001	India	Registered

NA: Not available, Mfg date: Manufacturing date, Exp date: Expiring date.

The results of the visual inspection as recorded in Table 1 revealed that three brands P004, P011 and P014 were not registered with the Pharmacy Board of Sierra Leone, highlighting the sales of unregistered paracetamol in the market. A similar study conducted on the quality assessment of Ascorbic acid sold in Freetown by Lahai M. et al. revealed that two out of the nine samples were not registered with the Pharmacy Board of Sierra Leone [23]. This suggests the need for the National Drug Regulatory Authority to continuously monitor the importers of these products to ensure their registration before making them available to the public.

Two brands P002 and P011 did not have manufacturing date labels, which makes their shelf life uncertain. This is similar to a study conducted in Katsina Metropolis Nigeria where 3 of the evaluated paracetamol brands did not have manufacturing or expiring dates [24]. Proper labelling is an important aspect of quality assurance of pharmaceutical products that provides essential information about the drug, such as its identity, strength, manufacturing and expiring dates, dosage form, directions for use, precautions, warnings, storage conditions and manufacturer's details [25].

All the other brands had full details of names and batch numbers, with manufacturing and expiry dates. They were all in their original packages as distributed to the various pharmaceutical outlets.

### 3.2 Assay of paracetamol tablets and identity test

The assay of paracetamol tablets was performed to determine the percentage content of paracetamol active ingredient in each sample; while the identity test confirmed the presence of paracetamol in each sample. The result of the assay tests is comprehensively outlined in Table 2. The percentage assay across the different brands ranged from 96.17 % (P008) to 101.97 % (P003), aligning with the accepted range of 95 %–105 % w/w as stated by BP Vol. 3 (2017).

**Table 2:** Assay and Uniformity of dosage units test results of different brands of paracetamol tablets.

Sample	Average percentage content from assay %±SD	Assay Limit % BP Vol. 3 (2017)	Calculated Acceptance Value AV ±SD	Acceptance Value (AV) - B.P Volume 5, 2017
P001	100.49 ± 0.20	95.00–105.00	1.98 ± 0.83	≤15
P002	98.44 ± 0.13	95.00–105.00	3.06 ± 1.25	≤15
P003	101.97 ± 0.02	95.00–105.00	3.45 ± 1.24	≤15
P004	101.33 ± 0.80	95.00–105.00	5.43 ± 2.26	≤15
P005	100.35 ± 0.16	95.00–105.00	3.59 ± 1.49	≤15
P006	99.37 ± 0.06	95.00–105.00	4.99 ± 2.08	≤15
P007	97.27 ± 0.06	95.00–105.00	3.95 ± 1.14	≤15
P008	96.17 ± 0.15	95.00–105.00	7.12 ± 1.20	≤15
P009	98.94 ± 0.55	95.00–105.00	7.43 ± 3.10	≤15
P010	100.55 ± 1.28	95.00–105.00	1.89 ± 0.78	≤15
P011	97.78 ± 0.90	95.00–105.00	3.78 ± 1.28	≤15
P012	98.81 ± 0.02	95.00–105.00	1.51 ± 0.63	≤15
P013	100.53 ± 0.59	95.00–105.00	7.64 ± 3.18	≤15
P014	97.55 ± 3.59	95.00–105.00	5.16 ± 1.75	≤15
P015	101.16 ± 1.22	95.00–105.00	5.00 ± 2.08	≤15

In identity test I, a violet-blue colour was observed in all the samples, indicating the presence of paracetamol. The test was repeated using the identity test II method, in which all the brands exhibited a violet colour that was gradually produced and did not turn red. This collective evidence underscores that all paracetamol brands evaluated in this study contained the requisite paracetamol content as their active ingredient.

Comparably, this is similar to a study done at Sana'a University, Republic of Yemen, where all the samples passed their Assay test, yielding an Assay range of 97.25 %–99.25 % [26]. This is also similar to another study conducted in Bangladesh in 2018, where similar testing revealed that all examined samples aligned with the specified range [27].

### 3.3 Uniformity of dosage units (mass variation)

One of the two approaches for demonstrating the consistency of dosage units as stated by BP Vol. 5 (2017) is mass variation. Each unit in a batch should have a drug substance content within a specified range as labelled on the drug [28]. This is calculated as Acceptance Value (AV).

The AV of the mass variation was calculated using the result of the assay and the standard deviation of the weighed sample as outlined earlier.

The detailed result of the uniformity dosage unit test is presented in Table 2. The different brands yielded an Acceptance Value ranging from 1.51 % (P012) to 7.64 % (P013). Remarkably, all the evaluated brands adhered to the acceptance value limit of ≤15, a standard stipulated by BP Vol. 5 (2017), indicating each dosage unit consistently contains the designated quantity of the drug substance within the predetermined range [29].

### 3.4 Dissolution of paracetamol tablets

The dissolution of paracetamol tablets was performed to measure the percentage of paracetamol that dissolved within 45 min. This test is based on the principle that tablets must dissolve in the gastrointestinal tract to produce their effect [30].

The results of the dissolution test are shown in Table 3. The result of the dissolution test revealed that 13 out of the 15 brands of paracetamol tablets complied with the BP Vol 5, 2017 specification of not less than 80 % dissolved in 45 min.

**Table 3:** Dissolution rate of different brands of paracetamol tablets.

Samples	S_1_ ±SD	BP Vol 5, 2017 Limit for S_1_ in %	S2: An average of 12 units (S_1_+S_2_) ± SD	BP Vol 5, 2017 Limit for S_2_ in %
P001	98.65 ± 3.55	NLT 80		
P002	97.72 ± 2.28	NLT 80		
P003	97.03 ± 1.68	NLT 80		
P004	102.34 ± 1.63	NLT 80		
P005	102.06 ± 4.38	NLT 80		
P006	82.95 ± 1.23	NLT 80		
P007	94.80 ± 3.57	NLT 80		
P008	91.31 ± 2.02	NLT 80		
P009	86.80 ± 3.75	NLT 80		
P010	85.18 ± 0.84	NLT 80		
P011	96.20 ± 1.05	NLT 80		
P012	19.22 ± 0.85	NLT 80	21.22 ± 5.76	Average of 12 units ≥ 75, and each unit NLT 60
P013	95.45 ± 3.26	NLT 80		
P014	65.97 ± 1.43	NLT 80	62.77 ± 9.53	Average of 12 units ≥ 75, and each unit NLT 60
P015	98.09 ± 0.94	NLT 80		

Value 1: S_1_ (Average percentage drug released in %); Value 2: S_2_ (Average percentage drug released in % done when sample fails stage 1). NLT: Not less than.

However, two of the brands (P012 and P014) failed the Stage 1 dissolution test with P012 releasing (21.22 % ± 5.76) of the drug within the stated time. The test was repeated for the two failed samples using S_2_ criteria but none of the samples passed. P014 with an average dissolution of 62.77 % ± 9.53, had some units less than Q-15 % (60 %) thus not meeting the BP Vol 5, 2017 criteria. This reflects the poor solubility of the two brands and possible low bioavailability when taken orally [31]. This result is contrary to a study conducted at Sana'a University; Republic of Yemen where all the different brands evaluated passed the dissolution test with a dissolution rate of 95.5 %–101.5 %. In a similar study done in Bangladesh in 2020, all the samples assessed passed their dissolution test [32].

The dissolution test is an important quality parameter that reflects the solubility, bioavailability and efficacy of oral drug products [31]. The dissolution rate depends on several factors, such as the formulation, manufacturing process, storage conditions, and physicochemical properties of the drug substance and the excipients [33]. The poor dissolution performance of some brands may be due to factors such as low solubility, high crystallinity, poor wettability, or inappropriate compression force [33,34]. Manufacturers of these brands with poor dissolutions should improve their formulation and process to ensure optimal dissolution rate and quality of their products.

### 3.5 Friability of tablets

Friability of tablets was performed to measure the tendency of tablets to chip, break or crumble after compression, physical shock or abrasion during manufacturing, packaging, shipping or handling [35,36]. It is usually applied to uncoated tablets. The results of the friability test are shown in Table 4. The different brands of paracetamol tablets tested gave a friability range of 0.09 % (P011) to 0.77 % (P015), which is a weight loss of less than 1 % w/w complying with the BP Volume V, 2017 specification for friability.

**Table 4:** Friability test results of different brands of paracetamol.

Samples	Initial weight (g) (W_1_)	Final weight (g) after Friability (W_2_)	Friability (%) Using Formula 2	BP Volume V, 2017 wt loss of less than 1 % w/w
P001	11.04	10.98	0.54	weight loss of <1 %
P002	11.82	11.80	0.17	weight loss of <1 %
P003	12.99	12.97	0.15	weight loss of <1 %
P004	13.47	13.43	0.30	weight loss of <1 %
P005	12.27	12.23	0.33	weight loss of <1 %
P006	13.89	13.85	0.29	weight loss of <1 %
P007	11.96	11.88	0.69	weight loss of <1 %
P008	12.07	12.04	0.25	weight loss of <1 %
P009	11.98	11.93	0.42	weight loss of <1 %
P010	11.42	11.40	0.18	weight loss of <1 %
P011	11.23	11.22	0.09	weight loss of <1 %
P012	12.35	12.32	0.24	weight loss of <1 %
P013	10.65	10.63	0.19	weight loss of <1 %
P014	12.55	12.53	0.16	weight loss of <1 %
P015	11.64	11.55	0.77	weight loss of <1 %

Hence, this result indicates that all 15 brands could withstand abrasion and external pressure from manufacturing, shipping and transportation without loss of tablet integrity. This is similar to a study conducted by Farhana et al. in Bangladesh where all samples passed the standard limit of friability but contrary to a study done in Somalia where two of the samples failed the friability test [35].

## 4 Conclusion

This study evaluated the quality of 15 brands of paracetamol tablets using Pharmacopoeia and Non-Pharmacopoeia tests. The study revealed that most of the brands conformed to the specifications for the percentage of active content, mass variation, friability and the uniformity of dosage units as stated by the BP. However, two of the brands did not have the correct label of manufacturing dates, making it difficult to determine their shelf-life. Two of the brands also failed the dissolution test parameter as stated by the BP, indicating that only a small amount of the drug would be released within the specified time, thus leading to subtherapeutic levels. Three of the brands were not registered with the Pharmacy Board of Sierra Leone. Our results highlighted the need for proper labelling, monitoring of registration status, and quality control of paracetamol tablets sold in Freetown to ensure their safety and efficacy for the consumers.

## Ethics statement

Permission to conduct the study was obtained from the research and innovation committee of the faculty of pharmaceutical sciences, College of Medicine, and the Allied Health Sciences University of Sierra Leone.

## Funding

Not applicable.

## Data availability statement

Most of the data is included in the manuscript. Additional data can be found from the corresponding author based on reasonable request.

## CRediT authorship contribution statement

**Ahmed Vandy:** Writing – review & editing, Writing – original draft, Methodology, Investigation, Data curation, Conceptualization. **Eugene Conteh:** Writing – review & editing, Visualization, Validation, Supervision, Methodology, Investigation, Formal analysis, Conceptualization. **Michael Lahai:** Writing – review & editing, Supervision, Methodology, Conceptualization. **Marie Kolipha-Kamara:** Writing – original draft, Methodology, Investigation, Formal analysis, Data curation. **Mohamed Marah:** Methodology, Investigation, Formal analysis, Data curation. **Foday Marah:** Writing – original draft, Investigation, Formal analysis, Data curation. **Kadiatu M. Suma:** Methodology, Data curation. **Sia C. Mattia:** Investigation, Formal analysis, Data curation. **Kenneth D.S. Tucker:** Investigation, Formal analysis, Data curation. **Victor S.E. Wray:** Investigation, Data curation. **Abass Koroma:** Investigation, Formal analysis, Data curation. **Aiah Lebbie:** Methodology, Investigation, Formal analysis, Data curation.

## Declaration of competing interest

The authors declare that they have no known competing financial interests or personal relationships that could have appeared to influence the work reported in this paper.
